# Work–Family Conflict and Turnover Intention Among Chinese Nurses: The Serial Mediating Roles of Career Identity and Work Engagement

**DOI:** 10.1155/jonm/3624666

**Published:** 2026-07-21

**Authors:** Meiling Song, Lanjun Zhang, Yan Li, Yu Sun, Xiaofan Bu, Zhimin Dai, Wenxiu Li, Yu Zhang, Yuqi Zhang, Ting Pan, Tingshi Zheng, Meixia Zhou, Luyao Li

**Affiliations:** ^1^ Department of Orthopedics, Zhejiang Hospital, Hangzhou, China, zjhospital.com.cn; ^2^ Department of Computing, The Hong Kong Polytechnic University, Kowloon, Hong Kong, polyu.edu.hk

**Keywords:** career identity, China, nurses, turnover intention, work–family conflict, work engagement

## Abstract

**Background:**

High turnover intention among nurses is a major threat to the stability of the nursing workforce in China. Work–family conflict is a salient job demand that may be associated with nurses’ career identity, work engagement and intention to leave, but the relationships among these constructs remain insufficiently understood.

**Objective:**

To examine the association between work–family conflict and turnover intention among Chinese nurses and to explore whether career identity and work engagement mediate this association, separately and sequentially.

**Methods:**

A cross‐sectional survey was conducted among registered nurses in China. Work–family conflict, career identity, work engagement and turnover intention were assessed using validated self‐report scales. Path analysis with bootstrapped confidence intervals was used to estimate the total, direct and indirect associations between work–family conflict and turnover intention.

**Results:**

Work–family conflict showed a substantial positive total effect on turnover intention (*β* = 0.394, *p* < 0.001). The total indirect effect of work–family conflict on turnover intention was small but significant (*β* = 0.053, *p* = 0.031). Neither work engagement alone (work–family conflict ⟶ work engagement ⟶ turnover intention) nor career identity alone (work–family conflict ⟶ career identity ⟶ turnover intention) significantly mediated this relationship. In contrast, the sequential pathway from work–family conflict to turnover intention via both career identity and work engagement (work–family conflict ⟶ career identity ⟶ work engagement ⟶ turnover intention) was statistically significant (*β* = 0.043, *p* = 0.011). After accounting for these mediators, work–family conflict remained directly associated with turnover intention (*β* = 0.341, *p* < 0.001), indicating partial mediation.

**Conclusions:**

Work–family conflict was directly and indirectly associated with nurses’ turnover intention. This study extends Job Demands–Resources theory (JD–R) by identifying career identity as a potential upstream professional resource and work engagement as a downstream motivational state in the association between work–family conflict and turnover intention. Given the cross‐sectional design, the findings should be interpreted as evidence of theoretically grounded associations rather than causal relationships.

**Implication for Nursing Management:**

The findings suggest that organisational strategies aimed at reducing work–family conflict and strengthening professional resources may be relevant for supporting nurse retention. Nursing managers may consider optimising staffing and scheduling systems, developing structured career development pathways and fostering engagement‐supportive leadership practices to support workforce stability.

## 1. Introduction

Nurses are indispensable to health systems, and adequate nurse staffing is associated with better patient outcomes and lower mortality [1]. However, nurse shortages and high turnover rates remain pervasive problems worldwide [2, 3]. Turnover intention refers to the likelihood that nurses will leave their current organisation, which is the strongest cognitive precursor of turnover [4, 5]. It can lead to a decline in the quality of nursing care, jeopardise patient safety, lower customer service standards and weaken overall healthcare management [6]. Globally, a recent meta‐analysis of 75 studies including more than 3.3 million nurses from multiple regions estimated that 38.4% (95% CI 31.0–46.4) of nurses report turnover intention, suggesting that roughly two in five nurses worldwide are considering leaving their current positions [3]. In China, large‐scale cross‐sectional surveys have consistently reported moderate to high levels of turnover intention among hospital nurses, with reported prevalence generally ranging from about 42.8% to 73.33% [7–10]. Identifying modifiable psychological processes that drive nurses’ intention to leave is therefore a priority for nursing management [11].

Work–family conflict is a prominent stressor for nurses, who must balance demanding clinical work with substantial family responsibilities. It is typically conceptualised as an inter‐role conflict in which pressures from work and family are mutually incompatible, so that participation in one role is made more difficult by participation in the other [12]. Meta‐analytic evidence indicates that work–family conflict is moderately and positively associated with turnover intention among nurses and other workers, with a pooled effect size of 0.28 [13]. Consistent with these findings, Chinese hospital nurses have shown that higher work–family conflict predicts stronger turnover intention, partly via reduced job and life satisfaction, with perceived supervisor support buffering these adverse effects [14].

Work engagement is commonly defined as a positive, fulfilling, work‐related state of mind characterised by vigour, dedication and absorption [15]. Nurses with higher levels of work engagement are associated with higher quality of care, better patient outcomes and lower turnover intention [16, 17]. Recent studies have shown that work engagement mediates the association between job resources (e.g. decent work, resilience and professional calling) and turnover intention, highlighting engagement as a key mechanism linking positive work conditions to nurse retention [18–20].

Career identity in nursing refers to how nurses see themselves as members of the profession, integrating their values, motivations, competencies and perceptions of the social significance of nursing into a coherent sense of meaning, belonging and commitment to a nursing career. Nurses with stronger career identity tend to report higher job satisfaction and better career development, as well as lower turnover intention [21, 22]. Related research has also emphasised the importance of professional values and professional self‐concept in shaping nurses’ attitudes and caring behaviours, suggesting that nurses’ professional self‐perceptions are closely connected with how they engage in clinical practice [23–25]. In addition, recent multicentre research has demonstrated that higher career identity is positively associated with work engagement, indicating that nurses who more strongly identify with their career tend to be more energetically and psychologically engaged in their work [26].

The present study is grounded in the JD–R theory, which provides a useful framework for explaining why some nurses remain engaged despite high job demands [27]. According to JD–R, job demands (e.g. workload, emotional strain and work–family conflict) are aspects of the job that require sustained physical or psychological effort and are therefore associated with certain physiological and psychological costs, whereas job resources (e.g. autonomy, social support, opportunities for development and professional identity) are aspects of the job that help achieve work goals, reduce job demands and stimulate personal growth and work engagement [27–29]. The pathway of JD–R suggests that adequate resources can buffer the negative impact of demands and foster engagement, which in turn improves work outcomes and reduces turnover intention, whereas insufficient resources under high demands are more likely to result in strain and withdrawal intentions [30, 31].

**FIGURE 1:**
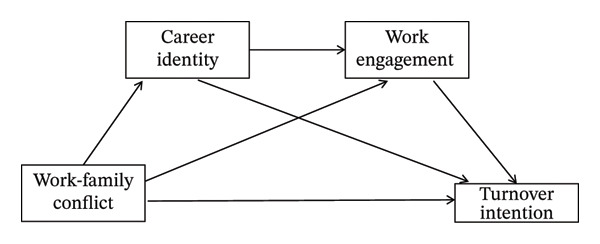
Proposed serial mediational model.

Although previous studies have examined the association between work–family conflict and turnover intention [32], professional identity and turnover intention [33] and work engagement and turnover intention among nurses [18], these constructs have often been investigated separately or as parallel mediating factors. This leaves an important gap in understanding how professional resources may operate sequentially in the pathway linking work–family conflict to turnover intention. In particular, limited attention has been paid to whether career identity functions as an upstream professional resource that connects work–family conflict with subsequent motivational processes, and whether work engagement serves as a downstream mechanism through which this resource pathway is associated with nurses’ intention to leave. Understanding these mechanisms may inform more precise targets for future interventions aimed at enhancing work engagement and retaining nursing staff, especially in settings such as China, where nurse shortages and high turnover intention remain pressing concerns [9]. Drawing on JD‐R theory, the present study conceptualises work–family conflict as a job demand, career identity as an upstream professional resource and work engagement as a downstream motivational state. By testing career identity and work engagement in one serial pathway, this study moves beyond examining isolated or parallel associations and helps clarify a theoretically grounded resource pathway from work–family conflict to turnover intention. Therefore, as shown in Figure 1, this study aimed to examine the direct association between work–family conflict and turnover intention among Chinese nurses and to test whether career identity and work engagement mediate this association separately and sequentially.

## 2. Methods

### 2.1. Study Design and Participants

This study employed a national multicentre cross‐sectional design. From June to July 2024, registered nurses were recruited across 22 provinces or autonomous regions in mainland China using convenience and snowball sampling. The research team first contacted nurses and managers through existing professional networks and personal contacts. These contact persons were informed about the study purpose, eligibility criteria and voluntary nature of participation, and were invited to distribute the survey link or QR code to eligible nurses in their hospitals and professional networks. Participants were also encouraged to forward the survey invitation to other eligible registered nurses, which helped extend recruitment across different provinces and regions. The participating nurses were recruited from tertiary, secondary and primary hospitals. Inclusion criteria were: (1) currently employed as a nurse in a clinical setting; (2) aged 18–60 years; (3) able to read and understand Chinese; and (4) willing to participate. Nurses with self‐reported mental disorders or other conditions impairing their ability to complete the questionnaire were excluded. Data were collected via an online platform (Questionnaire Star, https://www.wjx.cn/). Nurses accessed the anonymous questionnaire by scanning a QR code or clicking a survey link distributed by researchers. Electronic informed consent was obtained before questionnaire completion.

#### 2.1.1. Sample Size Determination

The required sample size was estimated using G∗Power software, Version 3.1.9.2. Because the proposed model was a path analysis based on observed composite scores, a linear multiple regression framework was used to approximate the sample size requirement. With an assumed effect size of *f* ^2^ = 0.10, a two‐sided alpha level of 0.05, statistical power of 0.90 and three predictors, the minimum required sample size was estimated to be 288 participants. Assuming a potential invalid response rate of 20%, the target sample size was increased to at least 360 participants.

### 2.2. Measures

#### 2.2.1. Work–Family Conflict

Work–family conflict was assessed using the Chinese version of the Work–Family Conflict Scale developed by Carlson et al. and adapted by Liu et al. [34, 35]. The instrument consists of eight items forming two subscales (work interferes with family and family interferes with work). Items are rated on a 5‐point Likert scale. Total score ranged from 8 to 40, with higher summed scores indicating greater levels of work–family conflict. Internal consistency was acceptable, with Cronbach’s *α* values of 0.80 for the family‐to‐work conflict subscale and 0.83 for the work‐to‐family conflict subscale [35]. In the present sample, Cronbach’s *α* for the overall scale was 0.858.

#### 2.2.2. Career Identity

Career identity was measured using the 21‐item Chinese version of the Nursing Career Identity Scale, which provides a multidimensional assessment of nurses’ sense of self‐efficacy and mastery, congruence, self‐determination, patient and organisational impact and meaningfulness [36]. Items are rated on a 7‐point Likert scale from 1 (*strongly disagree*) to 7 (*strongly agree*). Total score ranged from 21 to 147, with higher total scores reflecting stronger career identity. A previous study reported good internal consistency for the Chinese version (Cronbach’s *α* = 0.84) [36]. In the present sample, Cronbach’s *α* for the scales was 0.951.

#### 2.2.3. Work Engagement

Work engagement was assessed using the 9‐item Utrecht Work Engagement Scale (UWES‐9), which measures vigour, dedication and absorption [37]. Each item is rated on a 7‐point scale ranging from 0 (*never*) to 6 (*always*). Total scores range from 0 to 54, with higher scores indicating higher work engagement. The Chinese version of the UWES‐9 has shown satisfactory internal consistency (Cronbach’s *α* = 0.945) [38]. In the present sample, Cronbach’s *α* for the scales was 0.927.

#### 2.2.4. Turnover Intention

Turnover intention was measured using a six‐item nurse turnover intention scale that has been widely used in Chinese nursing research [21, 39]. The items capture three aspects: perceived likelihood of leaving the current job, motivation to seek alternative employment and perceived opportunities for obtaining other jobs. Each item is rated on a 4‐point Likert scale from 1 (never) to 4 (often); the total score ranged from 6 to 24, with higher total scores indicating stronger turnover intention. Cronbach’s *α* for this scale was 0.745 in a previous study [40]. In the present sample, Cronbach’s *α* for the scales was 0.826.

#### 2.2.5. Ethical Considerations

This study was approved by the Ethics Committee of Zhejiang Hospital (Approval No. 066K). Participation was voluntary and anonymous. Electronic informed consent was obtained at the beginning of the online questionnaire, and participants could withdraw at any time before submitting their responses.

#### 2.2.6. Data Quality Control

Several procedures were used to reduce duplicate and low‐quality responses. First, the online questionnaire was configured to allow only one submission from the same IP address. Second, all items were set as mandatory using the forced‐response function, so questionnaires with missing responses could not be submitted. Third, before data analysis, all submitted questionnaires were screened for eligibility and response quality. Responses were considered invalid and excluded if obvious low‐quality response patterns were identified, such as selecting the same response option for each scale. After data screening, all questionnaires were valid and included in the final analysis.

### 2.3. Statistical Analysis

All statistical analyses were performed using IBM SPSS 27.0 and Mplus 8, with a significance level of 5%. Descriptive statistics were used to summarise the sample characteristics and all the studied variables. Pearson’s correlation coefficient was utilised to examine the correlation between work engagement, work–family conflict, career identity and turnover intention. Path analyses were conducted in Mplus 8, using standardisation and bias‐corrected bootstrap 95% confidence intervals based on 5000 resamples. All variables were treated as observed continuous variables. A direct path from work–family conflict to turnover intention was included. The following indirect pathways from work–family conflict to turnover intention were examined: (1) via work engagement alone; (2) via career identity alone; and (3) via the sequential path career identity and work engagement. Standardised path coefficients, standard errors, *P* values and 95% confidence intervals were reported. An indirect effect was considered statistically significant if its 95% confidence interval did not include zero.

## 3. Results

### 3.1. Participant Characteristics and Mean Scores of the Study Variables

A total of 371 female registered nurses participated in this study. The mean age was 31.40 ± 6.41 years, and the average work experience was 8.78 ± 6.82 years. The overall mean scores were 23.09 for work engagement, 24.07 for work–family conflict, 105.02 for career identity and 15.76 for turnover intention. The largest proportion of participants worked 5–9 night shifts per month (42.59%) and were employed in tertiary hospitals (86.79%). The majority held junior professional titles (67.65%), had a bachelor’s degree (85.18%) and were contractually employed (51.75%). Descriptively, mean work engagement and career identity scores appeared higher among specialist nurses, nurses with senior professional titles, managers and those reporting job satisfaction. In contrast, higher turnover intention scores were observed among nurses with more frequent night shifts, contractual employment, lower job satisfaction and those without children. Details are shown in Table 1.

**TABLE 1:** Demographic and occupational characteristics of 371 nurses and mean scores of study variables.

Variable	Range/group	*N* (%)/Mean ± SD	Mean ± SD of study variables			
			Work engagement	Work–family conflict	Career identity	Turnover intention
Age	21–58	371 (31.40 ± 6.41)	23.09 ± 9.67	24.07 ± 6.01	105.02 ± 21.74	15.76 ± 4.05
Work experience (years)	0–35	371 (8.78 ± 6.82)				

Average number of night shifts per month (times)	0	52 (14.02%)	26.65 ± 10.77	23.98 ± 5.94	112.02 ± 19.74	14.40 ± 4.63
	1–4	108 (29.11%)	24.58 ± 9.75	23.20 ± 6.01	108.29 ± 22.78	15.36 ± 4.28
	5–9	158 (42.59%)	21.40 ± 8.92	24.42 ± 5.96	102.17 ± 21.52	16.30 ± 3.69
	≥ 10	53 (14.29%)	21.58 ± 9.33	24.91 ± 6.18	100.02 ± 19.83	16.32 ± 3.66

Average monthly income (¥)	≤ 5000	55 (14.82%)	24.31 ± 9.83	24.09 ± 5.90	103.40 ± 26.87	15.82 ± 4.20
	5000–10000	237 (63.88%)	22.43 ± 9.40	24.22 ± 6.13	103.70 ± 20.56	15.76 ± 4.00
	≥ 10,000	79 (21.29%)	24.23 ± 10.26	23.62 ± 5.79	110.11 ± 20.72	15.72 ± 4.15

As specialist nurses	Yes	92 (24.80%)	26.97 ± 10.64	24.59 ± 6.21	109.30 ± 23.61	15.28 ± 4.53
	No	279 (75.20%)	21.81 ± 8.98	23.90 ± 5.95	103.61 ± 20.94	15.92 ± 3.87

Professional title	Junior title	251 (67.65%)	21.29 ± 8.78	23.09 ± 6.01	101.73 ± 21.44	16.23 ± 3.68
	Intermediate title	109 (29.38%)	25.41 ± 9.42	24.22 ± 5.94	110.45 ± 20.54	14.86 ± 4.38
	Senior title	11 (2.96%)	41.00 ± 8.91	26.55 ± 6.70	126.55 ± 18.44	14.09 ± 6.53

Departments	Internal medicine	66 (17.79%)	20.15 ± 8.30	23.50 ± 5.60	103.74 ± 18.57	16.77 ± 3.31
	Surgery	114 (30.73%)	21.82 ± 10.07	23.84 ± 6.35	105.06 ± 24.42	15.72 ± 4.28
	Emergency	57 (15.36%)	21.81 ± 8.19	25.39 ± 5.83	98.21 ± 17.88	16.35 ± 3.52
	Gynaecology	83 (22.37%)	25.92 ± 9.82	23.66 ± 5.12	106.63 ± 22.51	15.00 ± 4.11
	Obstetrics	51 (13.75%)	26.57 ± 9.85	24.53 ± 7.18	111.61 ± 20.21	15.14 ± 4.57

As manager	Yes	24 (6.47%)	33.54 ± 10.63	26.00 ± 6.26	121.88 ± 17.71	13.88 ± 4.55
	No	347 (93.53%)	22.37 ± 9.18	23.94 ± 5.98	103.86 ± 21.53	15.89 ± 3.99

Hospital level	Tertiary hospitals	322 (86.79%)	22.70 ± 9.24	24.25 ± 6.01	104.14 ± 21.52	15.81 ± 4.02
	Secondary hospitals	36 (9.70%)	25.64 ± 10.96	22.53 ± 6.02	113.69 ± 23.41	15.50 ± 3.60
	Primary hospitals	13 (3.50%)	25.54 ± 14.78	24.08 ± 6.01	103.00 ± 18.29	15.23 ± 5.86

Mode of employment	Staffing of government–affiliated institutions	179 (48.25%)	24.02 ± 10.11	23.65 ± 6.22	107.73 ± 21.57	14.81 ± 4.40
	Contractual employment	192 (51.75%)	22.22 ± 9.18	24.47 ± 5.81	102.51 ± 21.65	16.65 ± 3.48

Job satisfactory	Unsatisfactory	53 (14.29%)	15.00 ± 10.24	27.21 ± 7.27	87.74 ± 23.15	18.68 ± 3.25
	General	240 (64.69%)	22.43 ± 7.39	24.50 ± 5.42	103.61 ± 19.40	16.21 ± 3.54
	Satisfactory	78 (21.02%)	30.60 ± 10.26	20.63 ± 5.24	121.13 ± 16.31	12.40 ± 3.86

With children	Yes	185 (49.87%)	24.83 ± 9.36	23.78 ± 5.63	110.15 ± 20.50	14.65 ± 4.31
	No	186 (50.13%)	21.35 ± 9.68	24.37 ± 6.37	99.93 ± 21.80	16.87 ± 3.44

Marital status	Single	138 (37.20%)	21.92 ± 9.76	24.72 ± 6.51	99.57 ± 22.66	16.99 ± 3.50
	Married	225 (60.65%)	23.61 ± 9.40	23.84 ± 5.59	108.02 ± 20.60	15.07 ± 4.10
	Divorced or widowed	8 (2.16%)	28.50 ± 13.30	19.38 ± 6.70	114.88 ± 19.02	14.13 ± 6.40

Education level	College or below	23 (6.20%)	24.30 ± 9.63	22.52 ± 5.36	105.78 ± 26.96	15.43 ± 3.85
	Bachelor degree	316 (85.18%)	22.72 ± 9.25	24.00 ± 5.90	105.11 ± 21.20	15.73 ± 3.97
	Master degree or above	32 (8.63%)	25.81 ± 13.06	25.88 ± 7.19	103.66 ± 23.55	16.34 ± 4.92

### 3.2. Descriptive Statistics

Table 2 shows the intercorrelations among the main study variables. Work engagement was significantly negatively correlated with work–family conflict (*r* = −0.138, *p* = 0.008) and turnover intention (*r* = −0.319, *p* < 0.001), and positively correlated with career identity (*r* = 0.607, *p* < 0.001). Work–family conflict was significantly negatively associated with career identity (*r* = −0.304, *p* < 0.001) and positively associated with turnover intention (*r* = 0.394, *p* < 0.001). Career identity was significantly negatively correlated with turnover intention (*r* = −0.314, *p* < 0.001).

**TABLE 2:** Correlations among the study variables.

Study variables	1	2	3	4
1. Work engagement	1			
2. Work–family conflict	−0.138^∗^	1		
3. Career identity	0.607^∗∗^	−0.304^∗∗^	1	
4. Turnover intention	−0.319^∗∗^	0.394^∗∗^	−0.314^∗∗^	1

^∗^ *p* < 0.05.

^∗∗^ *p* < 0.001.

### 3.3. Path Model and Mediation Analysis

**FIGURE 2:**
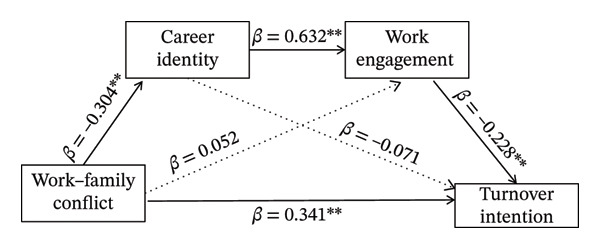
The serial multiple mediation analysis of the relationship between work–family conflict and turnover intention mediated via career identity and work engagement. *β*: standardised regression coefficients; ^∗∗^ *p* < 0.01.

The structural model with standardised path coefficients is presented in Figure 2, and the direct and indirect effects of work–family conflict on turnover intention are summarised in Table 3. Work–family conflict was negatively associated with career identity (*β* = −0.304, *p* < 0.001) and was not directly related to work engagement (*β* = 0.052, *p* = 0.366). Career identity, in turn, was positively associated with work engagement (*β* = 0.632, *p* < 0.001), and work engagement was negatively associated with turnover intention (*β* = −0.228, *p* = 0.002). The paths from career identity to turnover intention (*β* = −0.071, *p* = 0.307) and from work–family conflict to work engagement were not statistically significant. Work–family conflict had a significant positive direct effect on turnover intention (*β* = 0.341, *p* < 0.001).

**TABLE 3:** Standardised direct and indirect effects of work–family conflict on turnover intention.

Path	Standardised coefficient	SE	*p* value	95% CI
Total effect				
Work–family conflict ⟶ turnover intention	0.394	0.049	< 0.001^∗∗^	(0.294, 0.487)
Indirect effects				
1. Work–family conflict ⟶ work engagement ⟶ turnover intention	−0.012	0.014	0.414	(−0.052, 0.010)
2. Work–family conflict ⟶ career identity ⟶ turnover intention	0.022	0.021	0.310	(−0.020, 0.064)
3. Work–family conflict ⟶ career identity ⟶ work engagement ⟶ turnover intention	0.043	0.017	0.011^∗^	(0.016, 0.082)
Total indirect effect	0.053	0.025	0.031^∗^	(0.004, 0.103)
Direct effects				
Work–family conflict ⟶ turnover intention	0.341	0.049	< 0.001^∗∗^	(0.244, 0.435)

^∗^ *p* < 0.05.

^∗∗^ *p* < 0.001.

Table 3 presents the standardised total, direct and specific indirect effects of work–family conflict on turnover intention. The total indirect effect of work–family conflict on turnover intention was significant (*β* = 0.053, SE = 0.025, *p* = 0.031, 95% CI [0.004, 0.103]). When examining specific pathways, the indirect effects via work engagement alone (work–family conflict ⟶ work engagement ⟶ turnover intention) (*β* = −0.012, SE = 0.014, *p* = 0.414, 95% CI [−0.052, 0.010]) and via career identity alone (work–family conflict ⟶ career identity ⟶ turnover intention) were not significant (*β* = 0.022, SE = 0.021, *p* = 0.310, 95% CI [−0.020, 0.064] respectively). In contrast, the sequential indirect effect through career identity and work engagement (work–family conflict ⟶ career identity ⟶ work engagement ⟶ turnover intention) was statistically significant (*β* = 0.043, SE = 0.017, *p* = 0.011, 95% CI [0.016, 0.082]), indicating a meaningful chain‐mediating role of these two variables. After accounting for these mediators, work–family conflict still showed a significant positive direct effect on turnover intention (*β* = 0.341, SE = 0.049, *p* < 0.001, 95% CI [0.244, 0.435]), suggesting partial mediation.

## 4. Discussion

This study examined how work–family conflict is linked to turnover intention through career identity and work engagement. The findings showed that work–family conflict had a moderate, positive direct association with turnover intention, while also exerting a significant total indirect effect through career identity and work engagement. Work–family conflict was negatively associated with career identity, which in turn was positively associated with work engagement. Work engagement was negatively related to turnover intention, whereas the direct path from career identity to turnover intention was not significant after work engagement and work–family conflict were controlled. In addition, work–family conflict showed a significant serial indirect effect on turnover intention via lower career identity and reduced work engagement, while remaining a significant direct predictor of turnover intention, consistent with a partial mediation model. These results support a resource‐based explanation in which work‐family conflict is associated with lower professional resources and a stronger intention to leave. These findings deepen the application of the JD‐R theory in nursing workforce research by demonstrating that professional resources may operate in a sequential manner. Specifically, career identity appears to function as an upstream psychological resource that is associated with work engagement, which in turn is more proximally associated with turnover intention. Rather than acting as independent mediators, career identity and work engagement formed a connected resource pathway. This suggests that identity‐related resources may first influence the degree to which nurses invest themselves in their work, thereby influencing their retention‐related cognitions.

Importantly, these findings should be understood within the organisational context of nursing work. Work–family conflict is not only an individual experience, but also reflects organisational conditions such as rotating shifts, night and weekend work, workload pressure, staffing constraints and limited family‐supportive practices. From the perspective of JD‐R theory [27], these conditions represent job demands that may deplete nurses’ psychological resources and interfere with their professional functioning. In this study, higher work–family conflict was associated with lower career identity and, through reduced work engagement, stronger turnover intention. This pattern suggests that organisational conditions may shape nurses’ psychological functioning by weakening their professional identification and reducing their motivational investment in work. Given the cross‐sectional design, these findings should be interpreted as theoretically guided associations rather than evidence of causal relationships. Although the model was specified in a directional sequence based on JD‐R theory, the data do not allow causal inference or confirmation of temporal order among work–family conflict, career identity, work engagement and turnover intention.

### 4.1. Work–Family Conflict and Turnover Intention

The positive association between work–family conflict and turnover intention in this study is consistent with recent meta‐analytic evidence showing that work–family conflict is moderately and positively correlated with nurses’ turnover intention, with a correlation ranging from 0.15 to 0.47 (95% CI [0.02–0.66]) [13]. In China, nurses frequently work rotating shifts, nights and weekends, while at the same time carrying substantial family responsibilities, making work–family conflict a salient and persistent demand [26].

### 4.2. Career Identity, Work Engagement and the Serial Mediation Pathway

The model revealed a small but significant total indirect effect of work–family conflict on turnover intention (*β* = 0.053, *p* = 0.031), pointing to the presence of psychological mechanisms linking these constructs. Similar to our findings, previous studies have shown that the association between work–family conflict and nurses’ turnover intention is partly transmitted through attitudinal and affective processes, such as job and life satisfaction and positive/negative affect [14, 41]. Interestingly, neither work engagement alone nor career identity alone significantly mediated the association in our model. The indirect paths through work engagement (work–family conflict ⟶ work engagement ⟶ turnover intention) and career identity (work–family conflict ⟶ career identity ⟶ turnover intention) were both nonsignificant. This pattern suggests that simply reduced engagement or weakened career identity is not sufficient to account for the association; instead, more complex, multistep pathways are likely involved rather than a single mediator. The nonsignificant direct path from work–family conflict to work engagement may indicate that work–family conflict is not directly associated with nurses’ engagement once career identity is considered. In other words, work–family conflict may be more closely related to nurses’ professional self‐perception than to their immediate motivational state at work, and its association with work engagement may be expressed mainly through career identity.

The sequential indirect effect from work–family conflict to turnover intention through both career identity and work engagement was statistically significant (*β* = 0.043, *p* = 0.011). This serial mediation suggests that work–family conflict was associated with lower career identity, such as a weaker sense of meaning, belonging and commitment to the nursing career, which in turn was linked to lower work engagement and stronger turnover intention. Evidence from recent studies supports the idea of such connected resource pathways, for example, career identity and quality of work life jointly act as chain mediators between psychological resilience and work engagement in Chinese nurses [42]; and psychological capital and work engagement serially mediate the association between nurses’ well‐being and turnover intention [43]. Likewise, work engagement has been shown to mediate the relationship between decent work and turnover intention among registered nurses [18], and career identity and job burnout jointly form a chain‐mediating pathway from professional mission to turnover intention among operating room nurses [44]. Therefore, career identity and work engagement appear to function as a connected resource pathway. The nonsignificant direct path from career identity to turnover intention may further suggest that career identity is associated with turnover intention mainly through work engagement rather than through an independent direct pathway. When nurses identify more strongly with their career, they may be more energetic and psychologically invested in their work, which is more proximally related to their intention to stay or leave.

## 5. Implications

The findings have several implications for nursing management and policy. First, the direct association between work–family conflict and turnover intention highlights the importance of addressing the organisational conditions that generate work–family strain. Interventions aimed at stabilising the nursing workforce should therefore go beyond individual coping strategies and target modifiable workplace factors, such as staffing adequacy, flexible and family‐friendly scheduling, excessive night work, supervisor support and organisational support for work–life balance. Previous research has also shown that higher work–family conflict is associated with stronger turnover intention among nurses [41]. Family‐friendly workplace policies and family supportive supervisor behaviours reduce work–family conflict and improve job satisfaction and health outcomes, supporting the relevance of these approaches to Chinese nurses [45].

Second, the identified roles of career identity and work engagement suggest that strengthening positive professional resources is another important avenue for intervention. Studies have shown that a stronger nursing career identity is linked to higher job satisfaction and lower turnover intention, and that job satisfaction can fully mediate the association between career identity and turnover intention [21]. Career identity is also positively related to work engagement and career success among Chinese nurses, underscoring its importance for long‐term retention and development [22, 26]. Initiatives such as mentoring programmes, structured career development pathways, recognition of nursing contributions, participatory decision‐making and opportunities for reflection on the meaning and value of nursing work have been shown to enhance career identity and reduce burnout among new nurses, and could help nurses maintain a strong career identity in clinical settings [46]. Enhancing job resources such as decent work and learning opportunities can further promote work engagement and, in turn, reduce turnover intention [18].

Finally, the serial mediation pattern implies that multicomponent interventions may be particularly effective. Programmes that simultaneously address work–family conflict, foster career identity and build engagement may produce larger and more durable reductions in turnover intention than interventions targeting only one of these elements. This aligns with recent work demonstrating that combinations of personal and job resources (e.g. resilience, career identity and quality of work life) can jointly enhance nurses’ work engagement through chain‐mediating pathways [42], and with the broader gain spiral literature indicating that job resources can trigger upward spirals of engagement and well‐being over time [30]. These findings support the design of integrated resource‐building strategies (e.g. improving decent work conditions, strengthening career identity and cultivating engagement) as a way to buffer the negative impact of work–family conflict and promote sustainable nurse retention.

## 6. Strengths and Limitations

This study has several strengths. It used a multicentre sample of Chinese nurses, employed validated instruments for work–family conflict, career identity, work engagement and turnover intention, and tested a theory‐driven structural model with bootstrapped confidence intervals for indirect effects. It also builds on recent multicentre work from China examining work engagement and its correlates among nurses, thereby extending the focus from engagement levels to mechanisms linking work–family conflict to turnover intention [26].

Several limitations should be acknowledged. First, the cross‐sectional design precludes causal inference. Although the hypothesised model was specified in a theoretically assumed direction based on JD‐R theory, the temporal order among work–family conflict, career identity, work engagement and turnover intention cannot be established. Longitudinal studies are needed to determine whether changes in work–family conflict precede changes in career identity, work engagement and turnover intention over time. Second, all variables were assessed by self‐report, raising the possibility of report bias. Future research could incorporate administrative records (e.g. actual turnover) and multisource data. Third, the sample was recruited using convenience and snowball sampling through professional networks and personal contacts, which may have introduced selection bias. Although nurses were recruited from 22 provinces or autonomous regions in mainland China and from different hospital levels, the sample was heavily weighted towards tertiary hospitals, with relatively fewer participants from secondary and primary hospitals. Therefore, the sample should not be considered statistically representative of the general nursing population in mainland China, and the findings should be generalised with caution. In addition, because the study was conducted in mainland China and all participants were female, the generalisability of the findings to other countries, health‐care systems and male nurses may be limited. Finally, although this study collected several socio‐demographic and occupational characteristics of nurses, other relevant organisational, family‐related and cultural‐contextual factors were not included in the present model. For example, organisational factors such as leadership style, staffing adequacy and workplace support, as well as family‐related factors such as spouses’ employment status, caregiving responsibilities, household support and cultural expectations regarding family roles, may influence nurses’ work–family conflict and turnover intention. The omission of these variables may have limited the comprehensiveness of the model. Future studies should incorporate these broader contextual factors to provide a more complete understanding of the mechanisms underlying nurses’ turnover intention.

## 7. Conclusions

Work–family conflict was positively associated with turnover intention and negatively associated with career identity. Career identity, in turn, was positively associated with work engagement, and work engagement was negatively associated with turnover intention. Work–family conflict exerted a significant serial indirect effect on turnover intention through career identity and work engagement, while retaining a direct effect, supporting a partial mediation model. These findings underscore the importance of addressing both work–family conflict and positive professional resources when designing strategies to reduce turnover intention and improve nurse retention.

## Author Contributions

Meiling Song: conceptualisation, methodology, data collection, data curation, formal analysis, writing. Lanjun Zhang, Yan Li, Yu Sun, Xiaofan Bu, Zhimin Dai, Wenxiu Li, Yu Zhang, Yuqi Zhang, Ting Pan, Tingshi Zheng, Meixia Zhou: conceptualisation, data collection and writing. Luyao Li: conceptualisation, methodology, data collection, formal analysis and writing.

## Funding

The authors have nothing to report.

## Ethics Statement

This study was approved by the Ethics Committee of Zhejiang Hospital (Approval No. 066K).

## Consent

Informed consent was obtained from all the subjects through an online questionnaire. Participants were also informed that they had the option to withdraw at any time. All methods in this study were carried out in accordance with relevant guidelines and regulations.

## Conflicts of Interest

The authors declare no conflicts of interest.

## Data Availability

The data that support the findings of this study are available from the first author upon reasonable request.
